# Are we failing young people not in employment, education or training (NEETs)? A systematic review and meta-analysis of re-engagement interventions

**DOI:** 10.1186/s13643-016-0394-2

**Published:** 2017-01-25

**Authors:** Lauren Mawn, Emily J. Oliver, Nasima Akhter, Clare L. Bambra, Carole Torgerson, Chris Bridle, Helen J. Stain

**Affiliations:** 1 School of Psychology, Newcastle University, Newcastle upon Tyne, NE1 7RU UK; 2 School of Applied Social Sciences, Durham University, Durham, DH1 3HN UK; 3 Wolfson Research Institute of Health and Wellbeing, Durham University, Durham, TS17 6BG UK; 4 Institute of health and Society, Faculty of Medical Sciences, Newcastle University, Newcastle upon Tyne, UK; 5 School of Education, Durham University, Durham, DH1 3LE UK; 6 Institute of Health, Lincoln University, Lincoln, LN6 7TS UK; 7 Psychology - School of Social and Health Sciences, Leeds Trinity University, Horsforth, Leeds, LS18 4HD UK

**Keywords:** Unemployment, Effectiveness, Education, Health, Wages

## Abstract

**Background:**

Youth comprise 40% of the world’s unemployed, a status associated with adverse wellbeing and social, health, and economic costs. This systematic review and meta-analysis review synthesises the literature on the effectiveness of interventions targeting young people not in employment, education, or training (NEET).

**Methods:**

Randomised and quasi-randomised trials with a concurrent or counterfactual control group and baseline equivalence are included. Cochrane collaboration tools are used to assess quality, and a narrative synthesis was undertaken. The primary outcome is employment; secondary outcomes were health, earnings, welfare receipt, and education.

**Results:**

Eighteen trials are included (9 experimental and 9 quasi-experimental), sample sizes range from 32 to 54,923. Interventions include social skills, vocational, or educational classroom-based training, counselling or one-to-one support, internships, placements, on-the-job or occupational training, financial incentives, case management, and individual support. Meta-analysis of three high-quality trials demonstrates a 4% (CI 0.0–0.7) difference between intervention and control groups on employment. Evidence for other outcomes lacks consistency; however, more intensive programmes increase employment and wages over the longer term.

**Conclusions:**

There is some evidence that intensive multi-component interventions effectively decrease unemployment amongst NEETs. The quality of current evidence is limited, leaving policy makers under-served when designing and implementing new programmes, and a vulnerable population neglected.

**Systematic review registration:**

PROSPERO CRD42014007535

**Electronic supplementary material:**

The online version of this article (doi:10.1186/s13643-016-0394-2) contains supplementary material, which is available to authorized users.

## Background

Most young people succeed in education and make a positive transition to the world of work. However, global youth unemployment is estimated at 13.1%, three times that of adult rates [1] and equating to nearly 75 million individuals. This is a challenge faced by many high-income countries especially since the financial crisis of 2007/2008, with rates of a specific subgroup of young people aged 16–24 years and not in employment, education, or training (NEET) reported at 23.4% in the European Union, 15.5% in the USA, 12.2% in Australia, and 22.2% in the UK. Further, global youth unemployment has increased by 3.4 million since 2007 [2] and rates of NEET individuals and those in vulnerable employment continue to rise [1].

NEET individuals result in substantial economic costs to each country. For example, in the UK, there were an estimated 943,000 identified NEETs in 2015, despite claims of an economic recovery [3]. For each of these young people, the average lifetime direct cost to the public sector is £56,500 and the wider resource cost to the economy, including lost output, is estimated at £104,300 [4]. As a population, this has been projected to potentially cost the UK up to £77 billion in lost taxes, public service costs, and associated impacts such as crime and poor health [5].

In addition to the societal costs of NEETs, there are of course stark effects on the individuals concerned. Social inclusion, health, and wellbeing are all negatively impacted by unemployment from young adulthood and throughout life [6–9]. Unemployment increases the likelihood of medical consultations, taking medication and admission to hospital, and increases the risk of mortality [10]. The risk for psychiatric disorders, substance use, and suicidal behaviour is also increased for unemployed persons [11].

Reducing youth unemployment has been, and remains, a policy priority in many high-income countries including the UK, USA, and in Europe. Over the past few decades, there have been a number of initiatives and programmes implemented. In the UK, there have been specific programmes for youth since the introduction of the 1983 Youth Training Scheme [12]. More recent programmes include the New Deal for Young People (1998 to 2002), which provided work placements, vocational training, job search, and curriculum vitae support, plus the Educational Maintenance Allowance (1999 to 2011) which paid 16–18-year-olds an allowance to remain in full-time education. Recently, the UK Government announced apprentice schemes whereby, three million apprenticeships will be created by 2020 [13]. Additionally, much recent welfare reform has targeted NEETs, restricting their entitlements to key out-of-work benefits (such as housing benefits) and making participation in welfare schemes compulsory with non-participation leading to benefit sanctions and loss of income [14]. Across the world, NEET young people are considered to face particular barriers including: a lack of work experience, poor qualifications, heightened employer uncertainty, and—by some policymakers—considered to represent certain negative typologies (e.g. poor work, lazy, quitters; cf. [15]). As such, specific programmes are considered to be a way of providing additional support for the challenges faced by this group.

Past and present interventions targeting the NEET population are diverse. Intervention approaches include educational (academic, basic, or social skills; advice and guidance: [16, 17]); vocational (work placements, career planning, volunteering: [18]); counselling or mentoring [18, 19]; or service-based (case management, monitoring). Given that education is the most important risk factor for the development of NEET status, educational interventions target not only this established deficit but also the increase of work-related skills, knowledge, and aspirations. Thus, education serves as both an outcome and as the pathway through which engagement in work is achieved. In the present review, we focus on employment as our primary outcome, and do not exclude interventions targeting education, but recognise that our focus is primarily on this former aspect of NEET status.

Interventions working with the unemployed target a wide range of mechanisms theorised to influence engagement and wellbeing, for example developing efficacy, attitudes and perceived social norms [20], or enhancing social support and coping strategies [21]. The relative effectiveness of these, and other, different intervention approaches, however, is not known.

A lack of rigorous trial designs in evaluations of potentially effective interventions, rapid fluctuations in political and economic climates, and a diverse research base contributed to by scientific, statutory, and voluntary organisations, are potential factors leading to the paucity of knowledge of the effectiveness of interventions. However, given the longstanding and ongoing development of programmes in this area, it is important that evidence of effectiveness is examined. The aim of this systematic review was to identify, synthesise, and evaluate experimental or quasi-experimental evidence of the effects of any interventions, on employment, attainment, behavioural and health-related for youth classified as not in education, employment, or training.

## Methods

The protocol for the review was published ([name deleted to maintain the integrity of the review process]) and registered with the PROSPERO database, and a PRISMA checklist is available as Additional file 1.

### Trial identification and search strategy

A standardised search strategy [22] was used to search English language papers from 1990 to present. We justify narrowing as our focus given that, first, the vast majority of scientific articles are published in English and comprehension of literature would potentially be compromised by translation. Second, we suggest that target interventions are best understood in a contemporary context, hence use of the conventional inclusion threshold consistent with previous topical reviews [23].

The following databases were searched in June 2014 (replicated in May 2016): Medline, Embase, PsycINFO, ERIC, EPPI-Centre (Bibliomap), Social Science Citation Index, British Education Index, Conference Proceedings Index, Dissertation Abstracts, Popline, and grey literature collections (e.g. GLADNET). This was supplemented with internet searching (e.g. Google Scholar), forward and backward citation tracking from systematic reviews and included trials, and contact with trial authors and research groups. In addition, aid organisations with an interest in the target population were approached for internal reports (e.g. Barnardo’s). Together, these approaches identified some relevant papers outside of our original search restrictions.

### Eligibility criteria

Eligibility criteria were constructed around population, intervention, comparison, and outcomes (PICO). The *population* of interest was young people aged between 16 and 24 years who were not in employment or education (or training) at the time of the intervention commencing. We included trials for which the mean sample age was between 16 and 24 years, and those that reported analyses for NEET subgroups where the total population contained NEET and non-NEET individuals. There were no restrictions placed on trial inclusion in regards to country of population. Given one of our aims was to identify the full range of interventions that have been trialled with this group, we had no restrictions by *intervention* type. Any intervention that was delivered to the NEET population was included, whether targeted solely at NEET individuals or targeted at a larger group of unemployed individuals but reporting effects on NEET individuals separately. In terms of *study designs*, only randomised or quasi-randomised (i.e. where the method of group allocation is not truly random, such as matching, or alternate allocation) controlled trials, with a concurrent control or comparison group (including usual treatment controls) were included. We were not interested in excluding at this stage on the basis of the nature of the control or comparison group. Where a quasi-randomised design was used, groups had to demonstrate baseline equivalence or a valid matching protocol. Pre/post, cross-sectional, and non-comparison group designs were excluded. The primary *outcome* was employment; secondary outcomes included earnings, welfare receipt, education, health, and other behaviours (e.g. drug use).

### Quality assurance

**Fig. 1:**
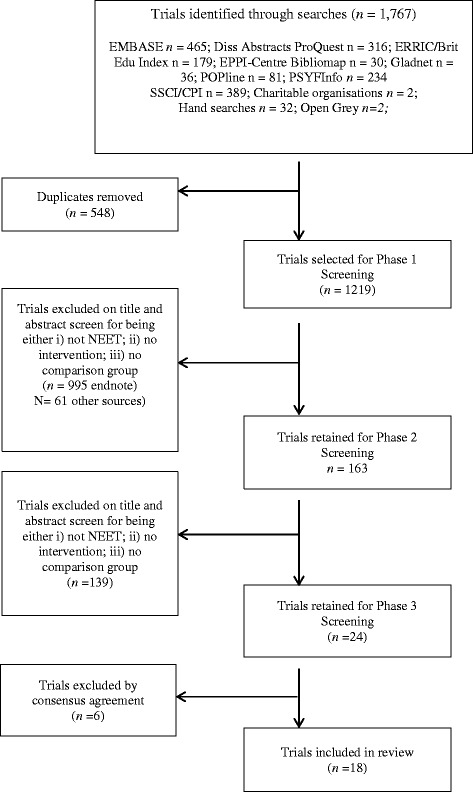
PRISMA flow chart

Search results were downloaded into Endnote. Following the removal of duplicate citations, a three-phase quality assurance process was conducted, using previously stated inclusion criteria. In phase 1, titles and abstracts were screened independently by two reviewers against the inclusion criteria. Agreement was high, with full consensus reached through discussion. To add rigour, 10% of trials excluded in this phase were cross-checked by a third author; no discrepancies emerged therefore we progressed to phase 2 screening. In phase 2, full text papers were again screened by two reviewers independently, with discrepancies resolved through discussion or, if necessary, by recourse to a third reviewer. Again, 10% of trials excluded in this phase were cross-checked with a third author; no discrepancies emerged and we progressed to phase 3. In phase 3, all papers were screened by a third author, and any disagreements resolved through group discussion ([initials deleted to maintain the integrity of the review process]). Search results, screening outcomes, and selection decisions are presented in a PRISMA flow chart in Fig. 1.

### Data extraction and quality assessment

Data were extracted using a standardised form, including methodological characteristics (e.g. unit of randomisation, length of follow-up), sample characteristics (e.g. prior length of NEET status), description of the intervention and control conditions (e.g. structure, theoretical basis, type, frequency, duration, provider and setting), measures and outcomes for baseline, and all follow-up periods and process-related outcomes (e.g. recruitment approach, uptake). The data extractions were completed by two authors ([initials deleted to maintain the integrity of the review process]) independently, cross-checked, and then quantitative extractions were verified by a researcher with statistical expertise ([initials deleted to maintain the integrity of the review process]). Where required data were missing, first or corresponding authors were contacted to request this information.

The assessment of trial quality and risk of bias was conducted independently by two authors ([initials deleted to maintain the integrity of the review process]) using the Cochrane Collaboration’s risk of bias assessment tool [24]. Each trial’s risk was rated as high, low, or unclear for sequence generation; allocation concealment; blinding of participants, assessors, and providers; selective outcome reporting; and incomplete data.

#### Data synthesis and statistical analyses

Summary measures of intervention effect size with associated estimates of precision (95% CI) were calculated for outcomes where minimal adequate data was available. There was insufficient quality of data to enable sub-group analyses, by either intervention type or participant characteristics. Where estimates could be extracted for sub-groups (e.g. males, females), these are reported separately. Meta-analysis was performed using a random effect model using post-intervention mean difference and standard error between intervention and control groups. There was insufficient data to consider statistical indicators of publication or small trial bias. Data were synthesised narratively by outcome.

### Deviations from protocol

Two important deviations from the protocol should be noted. First, not all of the stated analyses were conducted. Meta-analysis was only conducted on the primary outcome variable, employment. This was due to insufficient data reported within included trials for either meta-analysis or estimating publication bias. Mean difference was used as an effect measure instead of odds ratio for employment due to not having pre- and post data for intervention and control group for most trials. Second, due to the complexity and range of included analyses, an additional phase of quality assurance was conducted, with quantitative extractions reviewed by a researcher with statistical expertise ([initials deleted to maintain the integrity of the review process]).

## Results

### Trial flow

Of the 1767 citations identified, 1219 non-duplicate papers were retrieved. Nine hundred ninety-five were excluded in phase 1 screening (abstract), and 139 at phase 2 screening (full text) for not meeting the eligibility criteria. The most common rationale for exclusion was that the paper did not examine or report data for a NEET population. Six trials were removed following phase 3 screening (independent quality assurance). These included trials that used secondary data, and those with problematic control and/or for which baseline equivalence could not be established. Thus, 18 papers were retained: 13 journal articles, 3 reports (retrieved from ERIC, and 2 theses (see Table 1 for a summary of all included trials, including ID numbers). Of these trials that met the criteria for inclusion, nine were experimental randomised controlled trials (1, 2, 3, 4, 6, 7, 8, 15, 16) and nine were quasi-randomised (5, 9, 10, 11, 12, 13, 14, 17, 18). For two trials, subsample data that met the inclusion criteria were used (4, 6); and one author provided additional unpublished data for analysis.

**Table 1:** Characteristics of included trials

ID	Authors	Date	Design	Intervention	Control	Participant characteristics	Sample size	Outcomes measured	Effect size (*d*)
^1^	Alzua, Cruces, & Lopez-Erazo [30]	2013	Exp	Entra21 Classroom Internship Basic skills 884 h	No contact	Argentinian Below poverty line Unemployed 18–30 $\overline{x}\ \mathrm{age}$ = 23.55 (I), 23.80 (C) 33% male	407 randomised. 407 analysed.	Employment status Receipt of welfare Credit standing N credit enquiries	.154 – – –
^2^	Attanasio, Kugler, & Meghir [29]	2011	Exp	Jóvenes en Acción Classroom On-the-job 6 months, 5 h per day	Wait-list	Columbian Unemployed Lowest deciles of income distribution/Poor youth in urban areas $\overline{x}\ \mathrm{age}$ = 21.1 (I), 21.22 (C) 44.4%male	4353 randomised 3549 analysed.	Employment status Earnings	
^3^	Bloom, Orr, Cave, Bell, & Doolittle [33]	1993	Exp	JTPA II-A Classroom On-the-job Other services 3–5 months	Some received classroom training only.	USA Economically disadvantaged, facing barriers to employment. 45% male $\overline{x}\ \mathrm{age}$ = 19	4793 randomised. 4048 analysed.	Employment status Earnings Achieved HSD or GED	
^4^	Borland, Tseng, & Wilkins [31]	2013	Exp	YP^4^ Case management 23 meetings, 2 years	Standard service delivery	Australian Homeless (or history of homelessness/disadvantage) job seekers 18–35 $\overline{x}\ \mathrm{age}$ = 23.34 (I), 22.92 (C)	445 recruited. 208-355 analysed.	Employment status N days income support Receipt of welfare DEEWR programme expenditure Health and Wellbeing Community activities Housing	
^5^	Borland, & Tseng [34]	2007	Quasi	Job seekers diary Work search verification Fortnightly, 3 months	Standard service delivery	Australian 18–24	54,923 analysed (whole sample).	Receipt of welfare	
^6^	Card, Ibarrarán, Regalia, Rosas-Shady, & Soares [35]	2011	Exp	Juventud y Empleo Basic skills Internship 350 h	Not specified	Dominican Republic 44.5% male Lowest income members of working age population	5723 realised treatment group, 1623 realised control group.	Employment status Earnings	
^7^	Cave, Bos, Doolittle & Toussaint [28]	1993	Exp	Jobstart Basic skills Occupational training 800 h, 6.6 months	400 hours, not Jobstart.	USA Economically disadvantaged School drop-outs Low skilled 17-21 53.5% male	2312 randomised. 1491 analysed.	Employment status Earnings Receipt of welfare Receipt of education Receipt of qualification Pregnancy Criminal activity	
^8^	Chen [36]	2013	Exp	Job Corps Academic Vocational Social skills 8 months residential	Wait list (3 years)	USA Disadvantaged Low income household 16–24 $\overline{x}\ \mathrm{age}$ 18.42 (I), 18.38 (C)	15,386 analysed.	Employment status Earnings(weekly) Receipt of welfare	
^9^	Creed, Machin, & Hicks [37]	1996	Quasi	Youth Conservation Corps Work experience Classroom training 16 weeks	Wait list	Australian Unemployed >6 months 67% male (I), 52% (C) $\overline{x}\ \mathrm{age}$ = 18.76 (I), 18.71 (C)	245 randomised. 82 analysed.	Self-esteem Psyc. distress	
^10^	Creed, Machin, & Hicks [38]	1999	Quasi	Unnamed Work experience 6–12 months	Wait list	Australian 54% male $\overline{x}\ \mathrm{age}$ = 19 Unemployed ≥12 months Eligible for government sponsored programmes	65 randomised. 32 analysed at F3.	Psyc. distress Self-esteem	
^11^	Donovan, Oddy, Pardoe, & Ades [39]	1986	Quasi	Youth Opportunity Programme Work experience6–12 months	Did not access programme; unemployed.	UK $\overline{x}\ \mathrm{age}$ = 15.93 at T1 65% male	81 analysed.	Health status	
^12^	Stafford [40]	1982	Quasi	Youth Opportunity Programme Work experience 6–12 months	Did not access programme; unemployed.	UK 16–18 54% male	133 analysed.	Health (GHQ)	
^13^	Mounsey [41]	2002	Quasi	Youth training scheme Further education Placements On-the-job Duration not stated	No treatment; matched.	UK 16–17 at T1	Varied by analysis: 972 to 8885.	NEET status Expected earnings and reservation wages	
^14^	Nafilyan, & Speckesser [42]	2014	Quasi	Youth Contract Individually tailored support 12 months est.	Matched (counterfactual); same educational attainment and probability of receiving intervention.	UK 16–18	11,144 received intervention.	NEET status	
^15^	Schochet, McConnell, & Burghardt [43]	2003	Exp	Job Corps Educational Vocational Counselling Placements 8 months residential	Other services; not Job Corps.	USA Disadvantaged—living in a household that receives welfare or is below the poverty line, and living in an environment that impairs prospects for participating in other programmes. Free of serious behavioural and medical problems. 60% males>70% members of racial or ethnic minoritygroups 16–24	15, 406 randomised. 11,313 analysed.	Employment status Earnings	
^16^	Schochet, Burghardt, & Glazerman [44]	2001	Exp	Job Corps Educational Vocational Counselling Placements 8 months residential	Other services; not Job Corps.	As trial 15	15, 406 randomised. 11,313 analysed.	Employment status Earnings Receipt of welfare Receipt of education Health status Criminal activity	
^17^	Tanner, Purdon, D’Souza, & Finch [32]	2009	Quasi	Activity Agreement pilots One-to-one support Individually tailored contract Financial incentives 15 weeks	Standard service delivery; matched from non-participating areas.	England 58% males 16–17 NEET for >20 weeks Not receiving JSA.	1018 analysed at F1, 229 analysed at F2.	Education and employment Confidence and independence	
^18^	Grace & Gill [45]	2014	Quasi	YP^4^ Case management 23 meetings, 2 years	Standard service delivery	Australian Homeless (or history of homelessness/disadvantage) Job seekers 18–35	422 assigned, 370 analysed.	Earnings Welfare receipt Housing	

### Trial characteristics

The 18 included trials analysed between 122,488 and 131,337 participants (depending on outcome) with a median analysed sample size of 1 232, (range from 32 to 54, 923). The median of the mean ages was 19 years old (range = 15.93–23.67), of the trials that reported mean age (*n* = 8; 1, 2, 3, 4, 8, 9, 10, 11). Of the 12 trials that reported gender, percentage males in the sample ranged from 33 to 67%. The inclusion criteria varied across trials; please see Table 1 for full list of included trials and design, participants, location/country, intervention, and outcome characteristics.

The interventions reported in the trials included basic or social skills training (1, 6, 7, 8), vocational training (8, 15, 16), educational classroom-based training (1, 2, 3, 8, 9, 13, 15, 16), counselling or one-to-one support (15, 16, 17), internships, placements, work experience, on-the-job or occupational training (1, 2, 3, 6, 7, 9, 10, 11, 12, 13, 15, 16), financial incentives (17), work search verification (5), case management (4, 18), and individually tailored support (14, 17). The duration and intensity of interventions varied considerably. Three interventions lasted 12 months or more (4, 14, 18); nine lasted between 6 and 12 months (1, 2, 7, 8, 10, 11, 12, 15, 16); and five less than 6 months (3, 5, 6, 9, 17). The intensity of the interventions ranged from 23 sessions over a 2-year period to an 8-month full-time residential programme.

The control group interventions included no contact (1), standard service delivery (4, 5, 17, 18), use of other support services or restricted use of intervention programme services (3, 7, 15, 16), or placement on a wait list (2, 8, 9, 10). Matched data were used by four trials (11, 12, 13, 14), while one trial did not describe the control group/condition (6).

The outcomes measured were clustered in to six general domains: the primary outcome, employment; and secondary outcomes of earnings, welfare, education, health, and other. Twelve trials reported effects on employment status (1, 2, 3, 4, 6, 7, 8, 15, 16, 17), and two on NEET status specifically (13, 14). Eight trials reported effects on actual (2, 3, 6, 7, 8, 15, 16, 18) or expected (13) earnings. Seven trials reported receipt of welfare (e.g. income support; child support: 1, 4, 7, 8, 16, 18), and four trials reported either receipt of education (7, 16, 17) or educational attainment (3, 7). Health-related outcomes included general health status (5, 11, 12, 16) and psychological health (e.g. self-esteem, distress, confidence: 9, 10, 17). Other variables reported included credit standing (1), pregnancy rates (7), housing and community engagement (4, 18), health insurance provision (6), and criminal activity (7, 11, 16). Due to the diversity of outcomes, summary findings for these have not been collectively synthesised in this paper.

### Risk of bias

Risk of bias ratings for each trial (see Table 2) was examined using the Cochrane risk of bias tool [24]. Eight trials were at high risk of bias for sequence generation (4, 5, 6, 9, 10, 14, 16, 18), and the method of randomization was unclear in two trials (8, 15). For four trials, the risk of bias was not applicable due to matched counterfactual control groups (11, 12, 13, 17). Risk of bias owing to poor allocation concealment was high in five trials (4, 8, 14, 16, 18), not applicable in four trials (11, 12, 13, 17), and unclear in three trials (1, 10, 15). Lack of blinding created a high risk of bias for some outcomes in four trials (3, 6, 8, 17), was unclear in seven trials (4, 9, 10, 13, 14, 16, 18), and was not applicable to three trials (7, 11, 12).

**Table 2:** Risk of bias assessments for included trials

ID	Total classification	Sequence generation (selection bias)	Allocation concealment (selection bias)	Blinding (performance bias)	Outcome completeness	Selective outcome reporting	Other biases
1	High	+	?	+	+	-	-
2	Unclear	+	+	+	?	?	+
3	High	+	+	-	-	+	?
4	High	-	-	?	?	+	?
5	High	-	+	+	-	?	+
6	High	-	+	-	-	?	-
7	High	+	+	NA	-	+	+
8	High	?	-	-	+	-	?
9	High	-	-	?	?	-	+
10	High	-	?	?	+	-	?
11	High	NA	NA	NA	-	-	+
12	Unclear	NA	NA	NA	+	?	+
13	High	NA	NA	?	-	-	+
14	High	-	-	?	-	-	?
15	Unclear	?	?	+	?	?	+
16	High	-	-	?	-	-	?
17	High	NA	NA	-	-	+	?
18	High	-	-	?	?	?	?

+ low risk of bias; ? unclear risk of bias; - high risk of bias; NA not applicable

There was a high risk of bias due to incomplete outcome data for nine trials (3, 5, 6, 7, 11, 13, 14, 16, 17), and an unclear risk of bias for further five trials (2, 4, 9, 15, 18). This could be indicative of both a high rate of attrition in trials of this type of population and/or methodological deficiency in the trials themselves. Only four trials were clearly free of selective outcome reporting (3, 4, 7, 17), eight trials did not report all outcomes (1, 8, 9, 10, 11, 13, 14, 16), and it was unclear whether six trials reported all outcomes (2, 5, 6, 12, 15, 18). The quality of data reporting was also varied. For example, six trials reported means but not standard deviations (2, 3, 4, 7, 15, 16). Due to the small number of included trials, and small samples within some trials, we were unable to assess publication bias formally. Given that any additional unpublished trials could be sufficient to change estimates of the relative benefits and harms of these interventions, we considered that there was a high risk of publication bias.

### Main analysis

The findings are presented by outcome below (please see Table 3). Where possible, we have separated out findings by intervention type; however, this was challenging. All interventions featured direct contact with the population (i.e. none were indirect economic interventions). Most contained multiple elements (e.g. education, training and work placements, advice, support, and incentives); therefore, we were not able to create robust sub-groups by intervention type. The only meaningful division of interventions was comparing multi-component to single-component interventions. Even within these clusters, there was wide variation in terms of the intensity of delivery, rendering interpretation of effects based on intervention type problematic.

**Table 3:** Outcome data summary

ID	Authors	Outcomes measured	Effect size (*d*)	Mean difference (SE)	Comment
1	Alzua, Cruces, & Lopez-Erazo [30]	Employment status	.154	.113 (.049)	
		Receipt of welfare (F)	–	−.056 (.002)	Female only
		Credit standing	–	.524 (.813)	Sum of post treatment
		N credit enquiries	–	.900 (.342)	Sum of post treatment
2	Attanasio, Kugler, & Meghir [29]	Employment status (F)	.066	.054 (.022)	
		Employment status (M)	−.032	−.027 (.030)	
		Earnings (F)	.085	34668 (9743)	Columbian pesos
		Earnings (M)	.028	13690 (12819)	Columbian pesos
3	Bloom, Orr, Cave, Bell, & Doolittle [33]	Employment status (F)	–	2.8%	
		Employment status (M)	–	1.5%	
		Earnings (F)	–	−182	$USD
		Earnings (M)	–	−854	$USD
		Achieved HSD or GED (F)	–	5.8%	
		Achieved HSD or GED (M)	–	6.0%	
4	Borland, Tseng, & Wilkins [31]	Employment status	–	.03	No SE reported. 2-year follow-up.
		N days income support	–	18	3-year follow-up
		Receipt of welfare	–	267.2	$AUD; 3-year follow-up
		DEEWR expenditure	–	194.1	2-year follow-up
		Health	–	−.09	2-year follow-up; self reported
		Wellbeing	–	−.13	2-year follow-up; self reported
		Community activities	–	−.09	2-year follow-up; self reported
		Housing	–	−.05	2-year follow-up; self reported
5	Borland, & Tseng [34]	Receipt of welfare	–	−2.8	12-month follow-up; percentage chance in participants only (no control data)
6	Card, Ibarrarán, Regalia, Rosas-Shady, & Soares [35]	Employment status	.040	4.0% (3.9)	
		Earnings	.061	446 (284)	Dominican peso
7	Cave, Bos, Doolittle & Toussaint [28]	Employment status	–	.4%	Ever employed; 4-year follow-up totals:
		Earnings	–	214	$USD
		Receipt of welfare	–	−775	$USD
		Receipt of education	–	365.15	Hours in education
		Receipt of qualification	–	13.4%	
		Pregnancy	–	−4.9%	
		Criminal activity	–	−.3%	
8	Chen [36]	Employment status	.037	−.038 (.01)	
		Earnings(weekly)	.047	22.19 (4.65)	
		Receipt of welfare	−.021	−84.29 (38.27)	
9	Creed, Machin, & Hicks [37]	Self-esteem	.486	1.99 (4.14)	
		Psyc. distress	−.348	−1.93 (5.45)	
10	Creed, Machin, & Hicks [38]	Self-esteem	1.08	3.51 (3.05)	
		Psyc. distress	−1.43	−6.62 (4.25)	
11	Donovan, Oddy, Pardoe, & Ades [39]	Health status	–	−2.68 (.92)	Adjusted for T1 and gender
12	Stafford [40]	Health status	–	–	Cohort measured varied therefore comparison not possible
13	Mounsey [41]	NEET status:			Estimates using nearest neighbour matching
		YTS1 (M)	–	−.289 (.264)	
		YTS1 (F)	–	−.122 (.201)	
		YTS2 (M)	–	−.354 (.111)	
		YTS2 (F)	–	−.370 (.120)	
		YT (M)	–	.167 (.267)	
		YT (F)	–	.125 (.249)	
		Expected earnings	–	7.6%	
		reservation wages	–	8.6%	
14	Nafilyan, & Speckesser [42]	NEET status	–	−11.01	No SE presented
15	Schochet, McConnell, & Burghardt [43]	Employment status		2.9%	6.5-year follow-up
		Earnings	–	84	5.5-year follow-up; average earnings by quarter; $USD
16	Schochet, Burghardt, & Glazerman [44]	Employment status	–	3%	4-year follow-up
		Earnings	–	18.1	Average weekly earnings
		Receipt of welfare	–	−80.1	$USD
		Receipt of education	–	20.8%	Ever enrolled
		Health status	–	2.3%	Self-reported excellent
		Criminal activity	–	−3.8%	Ever arrested or charged
17	Tanner, Purdon, D’Souza, & Finch [32]	Education and employment	–	13.1%	
		Confidence	–	3.5%	Self report
		Independence	–	.6%	Self report
18	Grace & Gill [45]	Earnings	.025	1200	$AUD; 24-month follow-up
		Welfare receipt	.034	172	$AUD; 24-month follow-up
		Housing	.08	0.3	Stability: *n* of moves.

### Employment

**Fig. 2:**
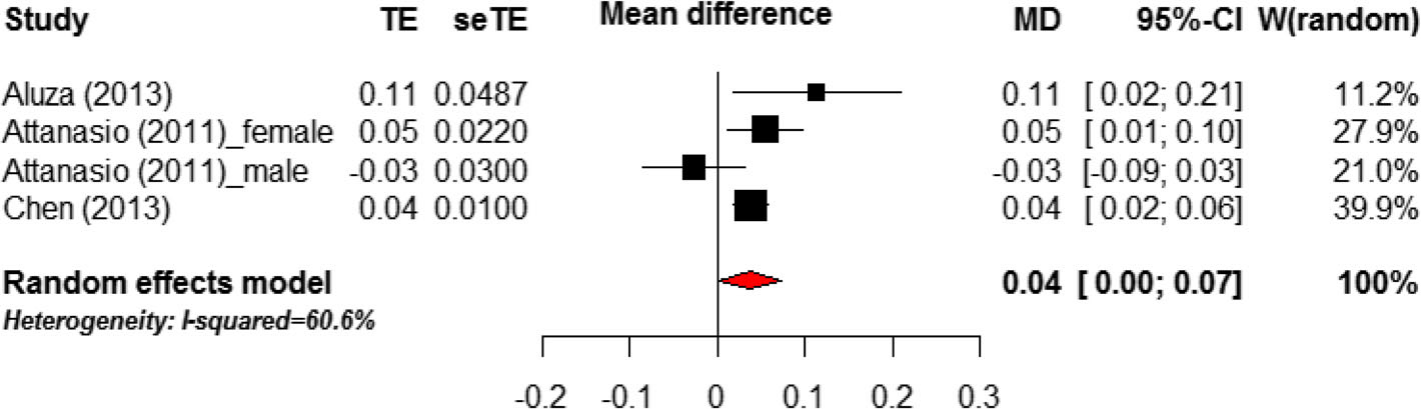
Meta-analysis of intervention effects on employment

Thirteen of the 17 trials reported employment or NEET status change as an outcome (1, 2, 3, 4, 5, 6, 7, 8, 13, 14, 15, 16, 17). Adequate data for meta-analysis (i.e. estimate of difference and standard error) was only available for four samples extracted from three trials (1, 2, 8). Post-intervention, the interventions had a small but significant positive effect on employment compared to control (MD = .04 [0.0–0.7]; see Fig. 2). It should be noted that follow-up periods varied from immediately post-intervention to 48 months. All three trials were multi-component interventions using a mixture of skills/educational training and job-based training.

Across all trials that reported employment as an outcome (including those meta-analysed above), nine were experimental and four were quasi-experimental designs, while the interventions used were heterogeneous (see Table 1 for trial characteristics). The majority (nine trials) used a multi-component intervention combining skills/educational training and job-based training. Of these, three had positive effects on employment (1, 8, 16) whereas one had positive effects for women only (2), four had no significant effect (3, 6, 7, 15), and one a negative effect (13). The only other multi-component trial combined one-to-one support with financial incentives (17) and had a significant positive effect.

In terms of the single component interventions, work search verification (5) had a positive impact on employment, whereas case management (4) and individually tailored support (14) had no effect. Across all 13 studies, commonalities of those with significant positive effects were inclusion criteria relating to deprivation indicators (e.g. below poverty line, lowest decile of household income); North or South American-based; post-2000; more likely to use multi-component interventions (e.g. classroom, job-based, and skills) and were for a minimum of 6 months of high intensity contact. Three of these four trials met data reporting requirements and were included in the meta-analysis.

### Earnings

Nine trials reported the effects on actual (2, 3, 6, 7, 8, 15, 16, 18) or expected (13) earnings. Meta-analysis could not be conducted for the outcome of earnings (three samples with sufficient data, three trials: 2, 8, 6), as precision estimates could not be calculated. All but one intervention was multi-component, featuring skills training (e.g. educational, vocational, basic, or social skills) combined with work-based learning (e.g. placement or internship). One involved trialling joined up case management (e.g. employment and housing service providers working cooperatively or collaboratively) (18). Apart from the case management trial, all were intensively delivered (one trial did not report intensity: 13), with a minimum of approximately 2.5 months of daily contact. Given this, analysis of effects by intervention type was not appropriate for this outcome, nor would intervention type explain differences in findings that emerged.

Three reported positive intervention effects on earnings. These were significant for one trial (8), significant for females only in another trial (2), and nonsignificant in one trial (6). Three trials, however, reported a more complex pattern of effects. In these trials, earnings for the intervention groups decreased in the first 2 years of participation but increased beyond the controls in the third and fourth year (7, 15, 16). Despite a common pattern, these differences were statistically significant in only one of the three trials. However, the magnitude of effect was generally small. One trial reported no significant intervention effect on earnings beyond the increase observed with standard provision (18).

There was some evidence suggesting effects on wages might manifest differently in different population subgroups. For example, one trial (3) found no effect on average earnings of female youths and a significant reduction for male youths of approximately $854 over 18 months. Another trial (8) identified stronger impacts on Hispanic participants when compared to whole sample data (U.S. population sample). Lastly, in one trial (7), earnings impact was stronger for those who chose to leave school due to disciplinary problems or dislike, as opposed to those who had left for employment-related reasons. Data for these claims was not included in the paper nor made available for re-examination on request. Of note, the trial that examined wage expectations (wage one expects to receive) and wage reservations (lowest pay one would consider) found a clear pattern of significant increases for men, but not for women.

### Welfare receipt

Six trials reported receipt of welfare outcomes (e.g. income support; child support: 1, 4, 7, 8, 16, 18). Only one trial (8) had adequate data when considering welfare receipt/benefits as the outcome and thus could not be meta-analysed. Two trials found significant intervention effects on receipt of public assistance, with reductions of $84.29/year (8) and $460 ID (16) across a 48-month follow-up period. Both were multi-component interventions, featuring skills training and work-based learning. The most minimal intervention (i.e. a change in case management procedures) had no significant effect on welfare receipt (4, 18).

Two trials reported significant differences in welfare receipt *only* for specific subgroups. One trial (1) of a multi-component intervention (skills and work-based training) had significant positive effect in credit use for male participants, and a significant negative effect on welfare dependency for females. Another (7) (skills and work-based training) found that the intervention reduced subsequent child-related welfare payments for women who were not custodial mothers on programme entry (relative to the control group), but not for women who were custodial mothers.

### Health

Health-related outcomes included general health status (4, 11, 12, 16), health behaviours (16), and psychological health indicators (9, 10). There were no trials with adequate data quality meta-analysis. Two interventions were multi-component (skills and work-based training), and four were single component (three work experience, one case management). The case management approach (4) resulted in no significant difference in health markers post-intervention. Of the work experience-only trials, two resulted in improved general health (11) or self-esteem and distress (10) and one poorer general health relative to the control groups (12). Of the multi-component trials, one had no significant effects on either health or health behaviours (e.g. alcohol, tobacco, or drug use: 16), and the other improved self-esteem but not psychological distress (9).

### Education

Four trials reported on either receipt of education or educational attainment (3, 7, 16, 17). There were no trials with adequate data quality for meta-analysis. All were multi-component interventions, and two different approaches were adopted. Three combined skills training and work-based learning, whereas one (17) offered individualised support and advice. Both approaches had positive effects. Three trials reported higher percentage of individuals receiving training for the intervention group compared to the control group (7, 16, 17). Two trials reported a ‘significant’ or ‘highly significant’ difference in General Education Development (GED) or High School Diploma (HSD) attainment (3, 7), and one trial a 7% increase in qualification attainment, for intervention groups compared to control groups (17).

## Discussion

### Summary of findings

This systematic review established the current state of evidence concerning the effectiveness of interventions targeting young people not in education, employment, or training (NEET). Based on the three trials with sufficient data to meta-analyses, the interventions resulted in a small but significant 4% increase in employment. Across the NEET population, this has the potential to enact change for thousands of individuals. Using conservative and somewhat crude estimates of costs and population [4], within the UK, this has the potential to equate to almost £469 million of savings to the public purse.

Successful interventions were high-contact (e.g. 884 h, 6 months, or an 8-month residential programme) and had additional commonalities in terms of inclusion criteria targeting deprivation and using multi-component approaches. Such interventions showed potential to result in small increases in earnings at longer-term follow-up (i.e. over 24 months) and reductions in welfare receipt, particularly for young women and those without children. No consistent effects on participants’ health were identified. However, there was evidence of increased educational attainment (for the most part, education would have been a direct consequence of intervention delivery). Although across all trials the majority of effects on employment were nonsignificant, it is important to note that the significant increase above emerged from synthesis of the highest data quality trials.

Taken together, the findings provide promising support for the effectiveness of high-contact multi-component (classroom and work-based) interventions in improving employment prospects for NEET individuals. These share some commonalities with effective practice highlighted in previous reviews. For example, the Department for Education [25] and Public Health England [26] highlighted the importance of work-based placements and basic skills provision and the involvement of local employers and accredited courses, respectively. Other reviews identify perceived important characteristics that are not supported in the current review (e.g. partnership arrangements, effective management and organisation, personalised learning, and clear progression routes: [27]).

We cannot, however, claim that high-contact multi-component interventions were universally effective, and interpreting the data at the intervention level is problematic in this review for multiple reasons. First, the vast majority of interventions were multi-component, combining some form of education or skills-based classroom training with on-the-job training (e.g. internship, work experience, job placements). It was notable that such interventions tended to adopt a pragmatic approach (e.g. classroom and work experience) rather than targeting potentially important psychological barriers to work engagement (e.g. enhancing confidence, reducing distress). Of note, narrative reviews have previously suggested that confidence-enhancing activities are beneficial [25]. Second, findings (both within and across outcomes) are mixed even when the same type of intervention is delivered. Third, there were insufficient number of trials available neither to compare different types of approach, in terms of content or modality (e.g. training versus job search modification), nor to examine required exposure to, or dose of, intervention necessary for a change in outcome.

A repeated finding of differential effectiveness for population sub groups is worthy of consideration here. Differences in intervention effects emerged in some trials dependent on gender, ethnicity, age, and broader circumstances (e.g. prior arrest rate). Sub-group differences were also reported in terms of recruitment to, and engagement with, interventions (e.g. [28]). Whilst the specific sub-groups more or less likely to benefit varied across trials and outcomes, it was notable that sub-groups benefitting less tended to be those that were more disadvantaged at trial commencement (e.g. poorer literacy, higher previous arrest rate, lower socioeconomic status, minority ethnic groups). This raises concerns that, despite often targeting a deprived population, current intervention approaches are not designed to cater for the circumstances and needs of the *most* disadvantaged, potentially further exacerbating the inequalities experienced by this group.

A more complex interpretation of subgroup effects emerged for gender differences, whereby, trials identified a significant effect on employment [29], a reduction in welfare receipt [30], and no short-term (i.e. <18 months) wage suppression [31] for females only. In these trials, females seemed to benefit more from the intervention, perhaps relating to lower levels of labour market engagement in general for young females relative to males in control populations (thus, improvements were more marked). Individual circumstances also seem to be important, for example, one trial identified reductions in welfare receipt for females who were not custodial mothers at trial commencement, but not for those who were. We tentatively suggest this is because non-custodial mothers were better placed to re-enter the labour market post-intervention, which implies that training alone is not sufficient to improve prospects for custodial mothers and perhaps psychosocial interventions could be beneficial.

Lastly, there was some evidence that contextual factors influenced intervention effectiveness. For example, Cave et al. [28] reported site level differences in effects and problems where different providers were responsible for different services. In trials where different methods of intervention delivery were compared, some reported similar impacts (with altered financial remuneration: [32]), some reported no differences in effect (e.g. between sequential versus simultaneous training delivery: [28]), and some reported different treatment effects (e.g. between variants of the YTS scheme: [33]). It should be noted that the trial locations (six countries, four continents), funders, and delivery partners varied; thus some interventions may have been effective due to the political and economic landscape of location and time of delivery.

### Research implications

Overall, the findings from the current review are inconsistent in respect to examining outcomes of interventions for NEET young people. We highlight five main areas for future research to address. First, there is still a need to establish *what works* to reengage young people. Notably, there is limited delivery and evaluation of interventions based on contemporary behaviour change theory and practice. Second, research is needed to establish what works *for whom*, particularly in light of interventions not serving some of the most disadvantaged. Third, it is not clear *what aspects* of interventions work (e.g. education and training, placement, counselling). Indeed, some arguably relevant approaches (e.g. psychological/behaviour change interventions) have not been subject to evaluation, therefore their potential impact is unknown. Fourth, there is a scarcity of research applying theoretically underpinned interventions. Fifth, there is a dearth of research examining physical and mental health outcomes, which is striking given the well established negative impact of unemployment on physical and mental health [6–9].

Previous narrative reviews of supporting young people who are NEET (e.g. [23]) have reported that ‘quality of the evidence is high, with most items based upon a strong to moderate evidence base that tends to be qualitative rather than based on statistical measurement’. We disagree. In contrast to this, our review not only found that there exists relevant work research utilising statistical measurement, but that the literature base has substantive issues with quality, methodological rigour, and reporting. For example, of note in the current research is the number of trials that did not provide sufficient data for inclusion in the meta-analysis. We recommend that high quality research is required and that trials evaluating effectiveness of interventions adhere to standardised reporting protocols (e.g. PRISMA) to aid future research examining the effectiveness of interventions with this population.

While methodological rigour is a challenge in terms of controlling for confounds (multiple agencies interacting with the population at any given time) and identification of an appropriate control group, there is a need to stress the importance of implementing randomised controlled trials so as to ascertain evidence for effectiveness and to ensure interventions are not having adverse effects (e.g. loss in earnings). Given that interventions are frequently delivered by commissioned private or voluntary organisations, there is a need for researchers to become involved early in programme development to aid with robust evaluations. Further, there are a broad range of providers and stakeholders working with NEET populations, including multiple local authority departments (e.g. housing, care, health), as well as international, national, and local aid organisations. The literature base reporting on interventions is therefore diverse, and useful information may be difficult to access (e.g. internal local authority project evaluations), incorporate, or control for. There are also systemic, cultural, and economic factors that are likely to impact on NEET status (e.g. recession, deprivation, policy, voting population). These make it difficult to eliminate all confounds when examining intervention effects, but in addition, highlight the importance of attention to these higher-level conditions when seeking to alter NEET population status.

### Policy implications

Reporting in terms of cost and cost effectiveness varied, and examining these was beyond the scope of the present review. It is worth noting, however, that intervention costs per recipient are low (e.g. $750 [29]; $1722 [30]). Although it is notoriously difficult to cost up the net social benefit of an individual moving from NEET to non-NEET status, where interventions are simple (e.g. embedded in existing services (5)) cost benefits were demonstrated. This is of mixed value to policymakers given that the strongest effects (i.e. on employment and earnings) emerge for the high-contact interventions.

When considering the commissioning and operation of high-contact schemes, we should be aware that the evidence identifies that the act of participation in such interventions may suppress earnings in the short term (within 24 months). Given this, schemes may need to consider financial incentives or wage replacement to improve recruitment and adherence rates. This may also assist with engagement within the interventions; in one trial [28] that reported effects segregated by contact, participants with low contact levels had poorer outcomes than the control group post-intervention, whereas those with high contact benefited greatly.

Public funders must recognise the need to support and fund rigorous trials as discussed above. Whilst recognising the desire to maximise access to services, this must not be at the expense of determining whether strategies are effective and cost-effective. In addition, limited funding should be allocated to programmes that will not contribute high quality evidence. Without this evidence, policy concerning how best to intervene is speculative. It is worthy of note that of the 18 included trials only three reported to be based on specific theories. One was driven by economic investment framework, and one was designed to increase job search efforts and matching to job vacancies as well as punitive monitoring and motivational feedback. Whilst a theoretical framework was not always explicitly articulated, there is an assumption that behaviourist theories underpinned both of these approaches. Finally, one trial utilised cognitive behavioural therapy-based training; however, this was aimed at improving the mental health of participants and providing them with coping skills to deal better with the negative consequences of prolonged unemployment rather than to reengage them in employment, per se. The limited use of explicitly theory-driven approaches to understanding and driving reengagement may have contributed to the limited variety of approaches utilised, and hence undermines our ability to identify what might work to reengage young people.

Considering most interventions aim to change participant behaviour, it is interesting to note that behavioural change theories were not employed more often. Potentially, this is an artefact of the dominance of economic and policy approaches to NEET interventions. To illustrate, there is a broad range of providers and stakeholders working with NEET populations, including multiple local authority departments as well as international, national, and local aid organisations. It may be that the NEET problem is being tackled by stakeholders focused on economics and social policy as opposed to those best placed to understand human behaviour change, disengagement and reengagement (e.g. psychologists, behaviour change specialists). Policy makers should consider engaging behaviour-change relevant expertise when designing intervention approaches.

As we still do not know how to effectively intervene to reengage NEET individuals, localised innovation should be promoted, accompanied by practice evaluations to identify nuances in delivery between sites and taking into account local contexts. Without effective interventions directly facilitating return to work or education, NEET individuals, and the countries that support them, are left exposed to fluctuations of the macro global economic climate. Good practice in terms of monitoring the NEET population should continue (e.g. within the UK, quarterly statistical releases are provided by the Department of Education), maintaining public and political momentum for tackling the issue. Technological approaches to service delivery and support, as well as monitoring, should be considered in the future as a potentially cost effective and accessible method for engaging this population.

### Limitations

This review included 18 trials and 131,707 participants. While this is not the first review examining the NEET population, other reviews (e.g. [23]) have not been restricted to experimental designs, instead including a broad range of trial methods. As a result, evidence included in these reviews is of limited use in terms of identifying effectiveness. Further, these reviews are prone to selective citation and lack robust quality assessment of included evidence, subsequently examining heterogeneity in a descriptive manner. The current review is the first, we believe, to enforce rigorous inclusion criteria relating to design as well as presenting robust quality appraisal processes.

We do recognise that by constraining the focus of this review to high-quality evidence we omit other work that may be important and useful. The learning from these service evaluations, qualitative trials, case trials, data analyses, models, and philosophical and theoretical texts should be considered holistically when debating the relative merits of different approaches to working with the NEET population.

We reviewed only robust evidence by restricting inclusion to randomised controlled trials and quasi-randomised trials with demonstrable baseline equivalence or a valid matching protocol. Despite this, concerns emerged when critiquing included trials against best scientific practice. All had a high or unclear risk of bias. We cannot know the extent or direction of the influence of bias on trials’ findings; however, under- or over-estimation of effects may be present. The ubiquitous nature of the bias risk also prohibited any additional analyses restricted to low risk trials.

As the interventions were all delivered in-service, over multiple sites, fidelity to experimental protocols would have been difficult to identify and were often not reported. We were unable to ascertain whether interventions were delivered as intended, in terms of either contact time or the nature of the provided contact. Where fidelity was reported, findings were not reassuring. For example, one trial [34] reported that 20% of their intervention group never received the intervention, and 50% had only one session in 6 months (as opposed to the targeted fortnightly administration). Concerns over fidelity were exacerbated in trials whereby control or comparison groups were also in receipt of an alternative intervention. For example, in one trial [35], control group members were transferred to intervention groups to compensate for individuals who did not attend the intervention.

## Conclusions

In a context where the number of youth classified as NEET is increasing globally and a priority area for labour market policy (International Labour Organisation, 2014; IMPETUS, 2014), identification of effective interventions is important. By considering a broad range of interventions and outcomes, this review has highlighted both gaps in the current evidence base, as well as examples of effective practice. Specifically, we have found that high intensity multi-component interventions, featuring classroom and job-based training, appear to increase employment amongst NEETs by 4% compared to controls. While it is disappointing to find that interventions appear to increase employment prospects by only 4%, it is important to acknowledge that in real terms this could represent a positive difference for thousands of young people. Further, importantly, although employment and earnings were the most commonly measured outcomes, some of the more promising findings emerged for mental health related outcomes. It may be that greater intervention effectiveness would be evident if wellbeing data were routinely monitored; indeed, theoretical questions regarding how we prioritise re-employment as opposed to targeting some of the pathways to re-employment and societal engagement more generally (including improved mental health) need attention from both researchers and policymakers.

However, more needs to be done to effectively meet the growing needs of the NEET population. Furthermore, considering the difficulty and cost of developing and delivering effective for NEET young people, there exists a critical need to do more to prevent individuals becoming NEET in the first place. Restrictions in the amount and quality of evidence leave us in a situation where best practice for changing the lives and prospects of NEET individuals for the better is unclear and robust future research is required. Whilst a key finding of this review was to highlight the need for future research to adopt high-quality evidence methodologies to determine what works best for this population, at present, limited recommendations for policy and practice can be endorsed. This leaves policy makers under-served when designing and implementing new programmes in this area, and a vulnerable population unacceptably neglected.

## Additional file

Additional file 1: PRISMA checklist. (DOC 63 kb)
